# Phototransduction in *Drosophila* Is Compromised by Gal4 Expression but not by InsP_3_ Receptor Knockdown or Mutation

**DOI:** 10.1523/ENEURO.0143-17.2017

**Published:** 2017-06-26

**Authors:** Murali K. Bollepalli, Marije E. Kuipers, Che-Hsiung Liu, Sabrina Asteriti, Roger C. Hardie

**Affiliations:** Department of Physiology Development and Neuroscience, Cambridge University, Cambridge, CB2 3EG, UK

**Keywords:** Gal4, GCaMP6F, GMR, phospholipase C, photoreceptors, TRP channels

## Abstract

*Drosophila* phototransduction is mediated by phospholipase C, leading to activation of transient receptor potential (TRP) and TRP-like (TRPL) channels by mechanisms that are unresolved. A role for InsP_3_ receptors (IP_3_Rs) had been excluded because IP_3_R mutants (*itpr*) appeared to have normal light responses; however, this was recently challenged by Kohn et al. (“Functional cooperation between the IP3 receptor and phospholipase C secures the high sensitivity to light of *Drosophila* photoreceptors in vivo,” *Journal of Neuroscience* 35:2530), who reported defects in phototransduction after IP_3_R-RNAi knockdown. They concluded that InsP_3_-induced Ca^2+^ release plays a critical role in facilitating channel activation, and that previous failure to detect *IP_3_R* phenotypes resulted from trace Ca^2+^ in electrodes substituting for InsP_3_-induced Ca^2+^ release. In an attempt to confirm this, we performed electroretinograms, whole-cell recordings, and GCaMP6f Ca^2+^ imaging from both IP_3_R-RNAi flies and *itpr*-null mutants. Like Kohn et al., we used *GMRGal4* to drive expression of *UAS-IP_3_R-RNAi*, but we also used controls expressing *GMRGal4* alone. We describe several *GMRGal4* phenotypes suggestive of compromised development, including reductions in sensitivity, dark noise, potassium currents, and cell size and capacitance, as well as extreme variations in sensitivity between cells. However, we found no effect of IP_3_R RNAi or mutation on photoreceptor responses or Ca^2+^ signals, indicating that the IP_3_R plays little or no role in *Drosophila* phototransduction.

## Significance Statement

Phototransduction in microvillar photoreceptors such as those of *Drosophila* is mediated by phospholipase C (PLC), culminating in activation of TRP channels, but how PLC is coupled to channel activation is unresolved. A recent study reported phototransduction defects after InsP_3_ receptor RNA interference (IP_3_R-RNAi), supporting a critical role for InsP_3_-induced Ca^2+^ release. However, we found that phototransduction was quantitatively unaffected not only after IP_3_R-RNAi, but also in IP_3_R-null mutants. Instead, we describe novel phenotypes in photoreceptors from flies expressing the transcription factor (Gal4) used to drive RNAi expression, which potentially account for the reported defects. The results indicate that IP_3_R plays no significant role in *Drosophila* phototransduction while emphasizing the need for caution when using Gal4 drivers.

## Introduction

Microvillar photoreceptors respond to light using G protein–coupled phospholipase C (PLC) cascades, leading to activation of nonselective cation channels (Yau and Hardie, 2009; Fain et al., 2010). In *Drosophila*, there are two such “light-sensitive” channels, encoded by the transient receptor potential (*trp*) and *trp*-like (*trpl*) genes. Both are permeable to Ca^2+^, with TRP being particularly Ca^2+^ selective (P_Ca_:P_Na_ ∼50:1) and contributing the majority of the light-induced current (Reuss et al., 1997; Liu et al., 2007). First cloned (Montell and Rubin, 1989) and identified as a light-sensitive channel (Hardie and Minke, 1992) more than 20 years ago, TRP is the prototypical member of the TRP ion channel superfamily, with 29 vertebrate isoforms distributed among 7 subfamilies. Of these, TRP and TRPL belong to and define the TRPC subfamily. All TRPCs can be activated via PLC; however, exactly how PLC activity leads to gating of the channels is unclear. Although InsP_3_-induced Ca^2+^ release is believed to be important for phototransduction in some microvillar photoreceptors (Brown et al., 1984; Fein et al., 1984; Ziegler and Walz, 1990; Walz and Baumann, 1995), a role in *Drosophila* had been excluded because light responses appeared to be unaffected in mutants of the InsP_3_ receptor (IP_3_R; Acharya et al., 1997; Raghu et al., 2000b). Subsequently, focus centered on other products of PLC activity such as diacylglycerol (Raghu et al., 2000a; Delgado et al., 2014) and its potential polyunsaturated fatty acid metabolites (Chyb et al., 1999; Leung et al., 2008; Lev et al., 2012), or PIP_2_ depletion and protons (Huang et al., 2010). Our own recent evidence suggested that the channels may be activated by a combination of protons released by the PLC reaction and the physico-mechanical consequences of cleaving PIP_2_’s bulky headgroup (InsP_3_) from the microvillar membrane (Hardie and Franze, 2012).

The conclusion that IP_3_Rs played no role in *Drosophila* phototransduction was seriously challenged by a recent study using RNA interference (Kohn et al., 2015). Those authors argued that previous failure to detect *IP_3_R* mutant phenotypes was due to leakage of trace Ca^2+^ from patch-clamp recording electrodes, effectively substituting for Ca^2+^ released from InsP_3_-sensitive stores. As supporting evidence, although light responses in *IP_3_R*-*RNAi* flies appeared normal in whole-cell recordings made without Ca^2+^ buffers in the electrode, they reported phenotypes using electrode solutions buffered with EGTA (Kohn et al., 2015). Phenotypes were also reported in electroretinogram (ERG) recordings suggesting a critical role *in vivo*. The authors proposed that InsP_3_-sensitive stores at the base of microvilli rapidly released Ca^2+^ into the microvilli and sensitized channels to activation via alternative products of PLC activity.

Because we found it surprising that Ca^2+^ leak could so effectively substitute for such a mechanism, we reinvestigated the role of the IP_3_R using not only RNAi but also IP_3_R-null mutants, paying particular attention to appropriate controls. In particular, Kohn et al. (2015) used the *Gal4*-*UAS* system (Brand and Perrimon, 1993) in which expression of *UAS-IP_3_R-RNAi* was driven by the *Gal4* transcription factor under the control of the strong eye-specific *GMR* promoter, but controls from flies expressing *GMRGal4* alone were lacking in most cases. We describe a number of novel phenotypes in flies expressing *GMRGal4*, but could not detect any differences in phototransduction between *IP_3_R-RNAi* flies or null *IP_3_R* mutants and relevant controls.

## Materials and Methods

Flies (*Drosophila melanogaster*) were reared in the dark at 25°C on standard medium (8.5 g cornmeal, 0.9 g agar, 1.5 g yeast, 7.5 g glucose, and 5 ml nipagin per 100 ml water). The wild-type strain was Oregon; for some experiments white-eyed mutants (*w^1118^*) were used, which are indistinguishable in terms of whole-cell electrophysiology, though more sensitive in ERG recordings.

Mutants and transgenic lines used included the following:

- *w^1118^*; *P{w^+^*,*GMRGal4*} (second chromosome, Bloomington stock 1104); referred to as *GMR*.
- *w^1118^; Sp*/*Cy*; *P{w^+^,GMRGal4},UAS*-*wRNAi* is referred to as *GMRw* (third chromosome); one copy of *GMRGal4*, *UAS-wRNAi* renders eye color almost white (very pale orange) despite presence of the wild-type *w* ^+^ gene or multiple mini *w* ^+^ transgenes (provided by A. Huber).
- *w^1118^*;;*P{w^+^ UAS*-*IP_3_R-RNAi*} (third chromosome VDRC stock 6486); homozygous stock appears wild-type in ERG and whole-cell recordings.
- *w^1118^;P{w^+^,UAS-GCaMP6f}* (second chromosome, Bloomington stock 47247).
- *w^1118^;P{w^+^,ninaE-GCaMP6f}* transgenic flies expressing GCaMP6f in photoreceptors R1-6 under control of the Rh1 opsin (*ninaE*) promoter: second and third chromosome lines made in-house using the pCaSpeR4 vector and GCaMP6f cDNA from Addgene (Asteriti et al., 2017).
- *norpA^H43^;bw;st* (white-eyed): expresses near normal PLC protein levels (80%) but has <10% catalytic activity due to a point mutation (Ser347Ala) in the catalytic site, and another (Thr1007Ser) in the C terminus (Yoon et al., 2004; obtained from B. Minke).
- *l(3)itpr^90B.0^* larval lethal, null mutation of IP_3_R due to small deletion; referred to as *itpr* (Venkatesh and Hasan, 1997); chromosome also has closely linked strong *P{w^+^}*.

To generate whole-eye IP_3_R-null *itpr* mosaics:

- *FRT82B*, *l(3)itpr^90B.0^/TM6:* (i.e., *itpr* recombined with *FRT82B*) were crossed to
- *yw;P{w^+^, ey-Gal4,UAS-FLP}/CyO;P{ry+,FRT82B}P{w^+^, GMR-hid},3CLR/TM6* (Bloomington stock 5253, referred to as *EGUF;FRT82B*). F1 non-*Cy* and non-*TM6* then have *itpr*-null mosaic eyes (Stowers and Schwarz, 1999; Raghu et al., 2000b).

To obtain similar eye pigmentation for ERGs, red-eyed (*w^+^*) *FRT82B, itpr/*TM6 flies were crossed to *yw*,*EGUF;FRT82B* flies and recordings made from red-eyed (*w^+^* males or *w^+^/yw* females) *itpr* mosaics, using sibling *itpr*/TM6, red-eyed wild-type, and wild-type mosaic eyes (by crossing to *w^+^;;FRT82B*) as controls. Further combinations using crosses from these parent lines are described in the text.

### Electrophysiology

Whole-cell patch clamp recordings of photoreceptors from dissociated ommatidia from newly eclosed, dark-reared adult flies of either sex were performed as previously described (e.g. Hardie et al., 2002) on inverted Nikon microscopes (Nikon UK). Standard bath contained (in mm): 120 NaCl, 5 KCl, 10 *N*-Tris-(hydroxymethyl)-methyl-2-amino-ethanesulphonic acid (TES), 4 MgCl_2_, 1.5 CaCl_2_, 25 proline, and 5 alanine, pH 7.15. For Ca^2+^ free solutions, CaCl_2_ was omitted and 1 mm Na_2_EGTA was added. Other solutions are described in text. The intracellular pipette solution was (in mm): 140 K gluconate, 10 TES, 4 Mg-ATP, 2 MgCl_2_, 1 NAD, and 0.4 Na-GTP, with or without 1 or 2 mm K_2_EGTA, pH 7.15. Chemicals were obtained from Sigma-Aldrich. Recordings were made at room temperature (21 ± 1°C) at –70 mV (including correction for –10-mV junction potential) using electrodes of resistance ∼10-15 MΩ. Series compensation of >80% was applied when required for macroscopic currents, but not for sampling quantum bumps and dark noise. Data were collected and analyzed using Axopatch 200 or HEKA amplifiers and pCLAMP v. 9 or 10 software (Molecular Devices). Quantum bumps and spontaneous dark events were analyzed using Minianalysis (Jaejin Software), analyzing typically at least 50–100 bumps/events per cell. Photoreceptors were stimulated via a green (540-nm) ultrabright light-emitting-diode (LED) controlled by a custom-made LED driver; intensities were calibrated in terms of effectively absorbed photons by counting quantum bumps at low intensities.

ERGs were recorded as described previously (e.g., Satoh et al., 2010) from flies of either sex immobilized with low-melting-point wax in truncated pipette tips using low-resistance (∼10 MΩ) glass microelectrodes filled with standard bath solution, one inserted into the eye and one into the head capsule near the ocelli. Light was delivered by an ultrabright red (640 nm) LED positioned within 5 mm of the eye. Although controls were always performed with flies having the closest possible eye color, the inclusion of variable numbers of P{*w* ^+^} transgenes (e.g., on *GMRGal4* or *UAS*-*RNAi* constructs as well as the *itpr* chromosome) meant it was not always possible to obtain strictly identical pigmentation. However, the use of red light minimizes any effect of variable eye color (note also that flies with two copies of *P{w^+^,UAS-IP_3_R-RNAi}* or two copies of the *itpr* chromosome would potentially have had darker eye colors than controls). The maximum intensity (10^0^ on figures) corresponded to ∼10^7^ effectively absorbed photons per photoreceptor per second in white-eyed wild-type flies (*w^1118^*). Signals were amplified by a DAM60 DC preamplifier (WPI) and sampled and analyzed using pClamp software (Molecular Devices).

### GCaMP6f measurements

Fluorescence measurements were made as previously described (Huang et al., 2010; Satoh et al., 2010; Asteriti et al., 2017) on an inverted Nikon microscope (non-confocal) from dissociated ommatidia or *in vivo* from intact flies via the deep pseudopupil (DPP). Excitation light (470 nm) was delivered from a blue power LED (Cairn Research), and fluorescence of whole ommatidia (via 40× oil objective) or DPP (20× air objective) was measured via a photomultiplier tube (Cairn Research) using 515 nm dichroic and OG515 longpass filters. Background fluorescence was subtracted using estimates from identical measurements from flies lacking fluorescent constructs. For dissociated ommatidia, Δ*F*/*F*_0_ was calculated using the *F*_0_ value measured in Ca^2+^-free solution (see above). To minimize any adverse long-term effects of exposure to Ca^2+^-free solution (e.g., depletion of Ca^2+^ stores) and hence to maximize the chance of detecting any Ca^2+^ release, ommatidia, plated in normal bath, were individually perfused by a nearby (∼10–20 µm) puffer pipette and measurements made within ∼20–40 s of perfusion onset, always confirming that a normal, rapid (latency <10 ms), and large fluorescence signal was recovered on return to normal (1.5 mm Ca^2+^) bath. The 1 or 2 s blue excitation light used to measure the fluorescence is a supersaturating stimulus and sufficiently bright to photoisomerize 100% of the visual pigment molecules at least once, reaching a photoequilibrium with ∼70% metarhodopsin. After the first measurement (usually made in Ca^2+^-free solution) the ommatidium was briefly exposed to intense, photoequilibrating red (4 s, 640 nm ultrabright LED) illumination to reconvert metarhodopsin to rhodopsin, returned to the control solution (1.5 mm Ca^2+^), and allowed to dark-adapt for at least 2 min before the next measurement. The effective intensity of the green illumination for *in vivo* measurements from the DPP was calibrated in effectively absorbed photons by measuring the rate at which it converted metarhodopsin to rhodopsin, as previously described (Hardie et al., 2015).

### Isolation of retinal tissue and PCR

Preparations of nearly pure *Drosophila* retinal tissue were collected as previously described (Matsumoto et al., 1982; Raghu et al., 2000b). Briefly, whole flies were snap-frozen in liquid nitrogen and dehydrated in acetone at –20° C for 4 d. The acetone was drained off, and retinae were separated as cleanly as possible using a flattened insect pin.

Total RNA was extracted by RNeasy Micro kit (Qiagen). Ten to twenty retinae were collected as described above for each biological group and homogenized by the TissueLyser (Qiagen) with six Zirconia 1 mm beads (Thistle) for 50 s twice, the samples were passed to the Qiashredder column (Qiagen), and standard procedures from RNeasy micro kit were followed. All nucleic acid preparations were quantitated by absorbance measurements at 260 nm using a NanoDrop instrument. The quantitative real time qRT-PCR was performed with a SuperScript III Platinum SYBR Green One-Step qRT-PCR kit (Invitrogen) and ABI 7500 fast instrument (Applied Biosystems). The levels of InsP_3_R transcript were analyzed using the following primers: 5′-GTGTGGCTCTTCACGGATCA-3′ (forward) and 5′-GAACTCCACCTTCGGAATCA-3′ (reverse). The housekeeping gene *Drosophila Ef1a48D* primers, TCCTCCGAGCCACCATACAG (forward) and GTCTTGCCGTCAGCGTTACC (reverse), were used as internal controls.

Quantitative PCR experiments on genomic DNA (gDNA) were also conducted using the ABI 7500 fast instrument. Ten retinae were collected for each biological group, and gDNA was extracted with 10 mm Tris, 1 mm EDTA, 25 mm NaCl, and 200 µg/ml fresh Proteinase K and homogenized by the TissueLyser with nine Zirconia 1-mm beads for 50 s four times. The samples were then incubated at 37°C for 30 min and 95°C for 3 min (to destroy the proteinase K). The qPCR was set up with Terra qPCR Direct SYBR Premix (Takara) alongside either the IP_3_R primers, 5′-AAAATGCGTAGCATCGCTCT-3′ (forward) and 5′-CACCACCGGCTTTAGTTGAT-3′ (reverse), or the primers from the *Rp132* housekeeping gene, 5′-CAAGAAGCTAGCCCAACCTG-3′ (forward) and 5′-CACTCACCGACAGCTTAGCA-3′ (reverse).

For verifying the presence of the *UAS-IP_3_R-RNAi* transgene, single flies were homogenized by the gDNA extraction buffer described above, and the following primers (sequences from VDRC) were used to perform the gDNA PCR: 5′-CGCGAATTCCCTTCGCCAGAGCGTGGAAA-3′ (forward), 5′-CGCTCTAGAACGCCAACATTGCGGAGCAG-3′ (reverse), and 5′-CACAGAAGTAAGGTTCCTTCACAAAGATCC-3′ (reverse).

### Statistical analysis

Statistical tests (two-tailed *t* tests or one-way ANOVAs with posttests as specified in the text and figure legends) were performed in GraphPad Prism5.

## Results

### *GMRGal4* phenotypes

The phenotypes reported by Kohn et al. (2015) came from flies in which IP_3_R expression was suppressed by *UAS-IP_3_R-RNAi* (two copies in most experiments), driven by one copy of *GMRGal4*. However, in most cases, controls from flies expressing *GMRGal4* alone (without *UAS-IP_3_R-RNAi*) were lacking. This is an important control, because *GMRGal4* homozygotes expressing two copies of *GMRGal4* have severe degeneration and developmental phenotypes (Kramer and Staveley, 2003), and although one copy of *GMRGal4* is often assumed to cause no phenotype, this has not been thoroughly explored. The *GMRGal4* line used by Kohn et al. (2015) had a second chromosome *GMRGal4* transgene recombined with *UAS*-*wRNAi*, which induces a white-eyed phenotype by suppressing expression of the wild-type *w* gene or the *w^+^* marker gene in various expression vectors (Kalidas and Smith, 2002). Unfortunately this line has been lost (B. Minke, personal communication); therefore we tested a line with the same *GMRGal4,UAS-wRNAi* combination inserted on the third chromosome (referred to subsequently as *GMRw*), as well as the second chromosome *GMRGal4* line (Bloomington stock 1104, referred to simply as *GMR*) but without *UAS-wRNAi.* We used the same *UAS*-*IP_3_R*-*RNAi* line as Kohn et al. (2015), VDRC stock 6486, confirming the presence of the *IP_3_R-RNAi* transgene in all the backgrounds used by gDNA PCR of diagnostic sequences (from VDRC website). Effective knockdown of IP_3_R mRNA was validated by qRT-PCR of dissected retinal tissue, with 32.8% ± 1.7% (mean ± SEM, *n* = 3 independent samples) IP_3_R mRNA remaining compared with *GMR*/+ controls. Approximately half of this (∼15%) is likely attributable to contaminating tissue in the dissected retinae (Raghu et al., 2000b).

**Figure 1.**
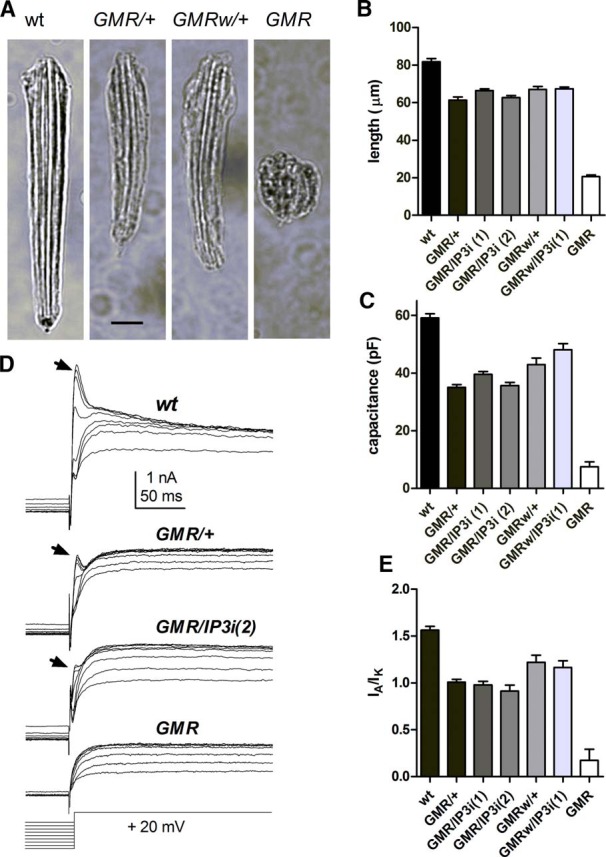
Morphologic and electrophysiological *GMRGal4* phenotypes. ***A***, Bright-field micrographs of dissociated ommatidia in wild-type and flies expressing one (*GMR*/+) or two (GMR) copies of *GMRGal4*. Scale bar, 10 µm. ***B***, Length of ommatidia in wild type and flies expressing one or two copies of *GMRGal4* with or without one or two copies of *UAS*-*IP_3_R*-*RNAi* (mean ± SEM, *n* > 10 randomly selected ommatidia from three to four flies per genotype). ***C***, Capacitances in whole-cell recordings from same genotypes (*n* = 8–34 cells per genotype). Both ommatidia length and capacitance in flies with one copy of GMRGal4 were significantly reduced compared with wild-type (*p* < 0.001, one-way ANOVA with Dunnett’s multiple comparison test). ***D***, Voltage-sensitive potassium currents at 20 mV after negative 1-s prepulses (–20 to –100 mV) to remove inactivation. Arrows indicate the rapidly inactivating *Shaker* component (I_A_), which was greatly reduced in flies expressing one copy of *GMRGal4* with or without *IP_3_R*-*RNAi* (two copies) and virtually absent in *GMR* homozygotes. After 100 ms, the remaining maintained current is largely mediated by delayed rectifier (*Shab* = I_K_) channels. ***E***, Ratio of I_A_ (*Shaker*) peak current to I_K_ (*Shab*) current measured 100 ms after voltage step: all backgrounds with one copy of *GMRGal4* show significantly reduced I_A_ compared with wild-type. Dunnett’s multiple comparison test, *p* < 0.003, *n* = 7–17 cells per genotype, except *GMRw*/*IP3R*-RNAi (*n* = 3).

**Figure 2.**
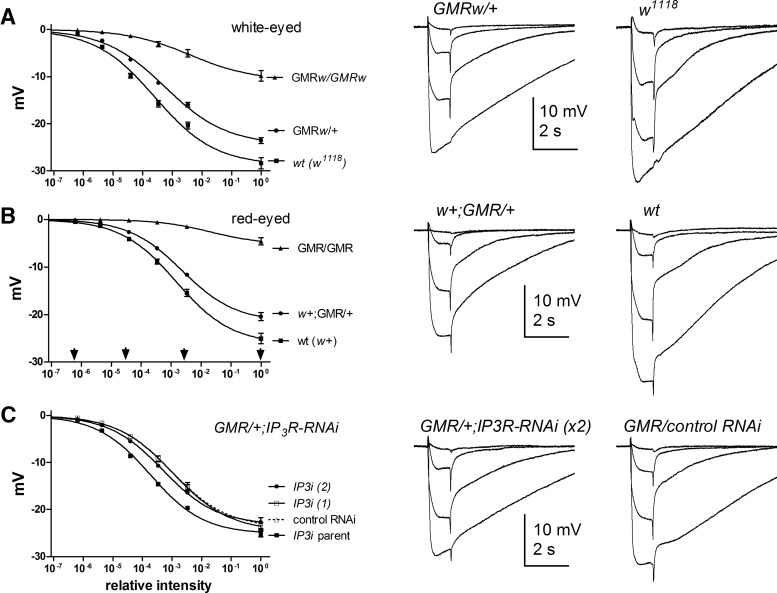
*GMRGal4* ERG phenotypes. Response intensity (*V*/log *I*) functions measured from ERG (plateau at end of 1-s stimuli). ERGs from flies with one copy of *GMRw* (***A***) or *GMR* (***B***) were significantly reduced in amplitude across all intensities compared with respective eye color–matched wild-type controls (mean ± SEM, *n* = 10–17 flies; *p* < 0.0001; two-tailed *t* tests). ***A***, *GMRw*/+ (F1 of *Sp/Cy;GMRw* × *w^1118^* compared with *w^1118^*). Data from *Sp/Cy;GMRw/GMRw* parent also plotted. Representative traces on right (at intensities marked by arrows in ***B***). ***B***, F1 of *w^+^;GMR* × wild-type (red-eyed) cross compared with red-eyed wild-type (w+) and *GMR/GMR* homozygote, *n* = 10–14 flies. ***C***, In contrast, ERGs of *GMR/+;IP_3_R-RNAi* (one copy, *n* = 16, or two copies, *n* = 19) were similar to a control *GMR*/+ (control RNAi; *n* = 10, dotted line). Flies were F1 of *GMRGal4*;*IP_3_R*-*RNAi*/TM6 × *UAS*-*IP_3_R*-*RNAi* or *UAS*-*fwd*-RNAi (control RNAi line chosen because it has similar eye color, but no effect on photoreceptor physiology). However, all *GMR*/+ genotypes were less sensitive (*p* < 0.0001) than the *IP_3_R*-*RNAi* parent stock (*n* = 7). Maximum intensity (10°) equivalent to ∼10^7^ effectively absorbed photons per photoreceptor in wild-type (*w^1118^*).

Homozygote flies carrying two copies of *GMRGal4* displayed severe defects in retinal morphology (Fig. 1) and physiology (Fig. 2). The surfaces of the eyes were “glassy” with irregular facets, and ERG responses were greatly reduced in amplitude (Fig. 2). Dissociated ommatidia were almost unrecognizable, presenting as roughly spherical clusters, similar to midpupal-stage (∼48-h) ommatidia before the rapid phase of elongation that results in the characteristic adult appearance (Fig. 1*A*). Eyes of flies expressing just one copy of *GMRGal4* appeared superficially normal; however, on preparing dissociated ommatidia from either *GMRGal4* line (with or without *IP_3_R-RNAi*), it was apparent that *GMRGal4*/+ photoreceptors did not have the usual wild-type adult appearance. Ommatidia with one copy of *GMRGal4*, irrespective of *IP_3_R*-*RNAi*, were shorter than in wild-type (∼65 vs. ∼85 µm) and often had a less well-formed appearance (Fig. 1*A*, *B*), now somewhat reminiscent of ommatidia prepared from late-stage pupae. In whole-cell recordings, cell capacitances—which largely reflect the area of microvillar membrane—were also significantly reduced in *GMRGal4/+* photoreceptors (∼30–50 pF vs. 50–70 pF), again irrespective of *IP_3_R*-*RNAi* (Fig. 1*C*). There were also marked differences in the voltage-sensitive potassium channel profiles (Fig. 1*D*, *E*). In wild-type photoreceptors, the largest component is a fast-inactivating A-current (I_A_) encoded by the *Shaker* gene, which typically reaches ∼4 nA (at 20 mV), vs. 2–3 nA for the delayed rectifier encoded by *Shab* (Hardie, 1991b; Vahasoyrinki et al., 2006). However, in *GMR*/+ and *GMRw*/+ flies, the *Shaker* component was substantially reduced, and in *GMR* homozygotes, almost undetectable (Fig. 1*D*, *E*). During photoreceptor development, the *Shaker* current appears last, only at late-pupal stages (Hardie, 1991b), so this profile is again suggestive of stunted development.

The ERG is a widely used indicator of *in vivo* photoreceptor performance, although it is a complex signal reflecting responses of photoreceptors, glia, and second-order neurons as well as extracellular resistance barriers (Heisenberg, 1971; Kohn and Minke, 2011). Kohn et al. (2015) reported that ERGs in *GMRGal4*/+;*IP_3_R*-*RNAi* flies were reduced compared with wild-type, but did not provide data from *GMRGal4*/+ controls. We found that ERGs recorded from *GMR*/+ and *GMRw/+* flies were significantly reduced in amplitude and sensitivity compared with eye color–matched wild-type controls. However, *GMR/+;IP_3_R-RNAi* with either one or two copies of *UAS-IP_3_R-RNAi* showed little or no difference from *GMR*/+ controls (Fig. 2).

**Figure 3.**
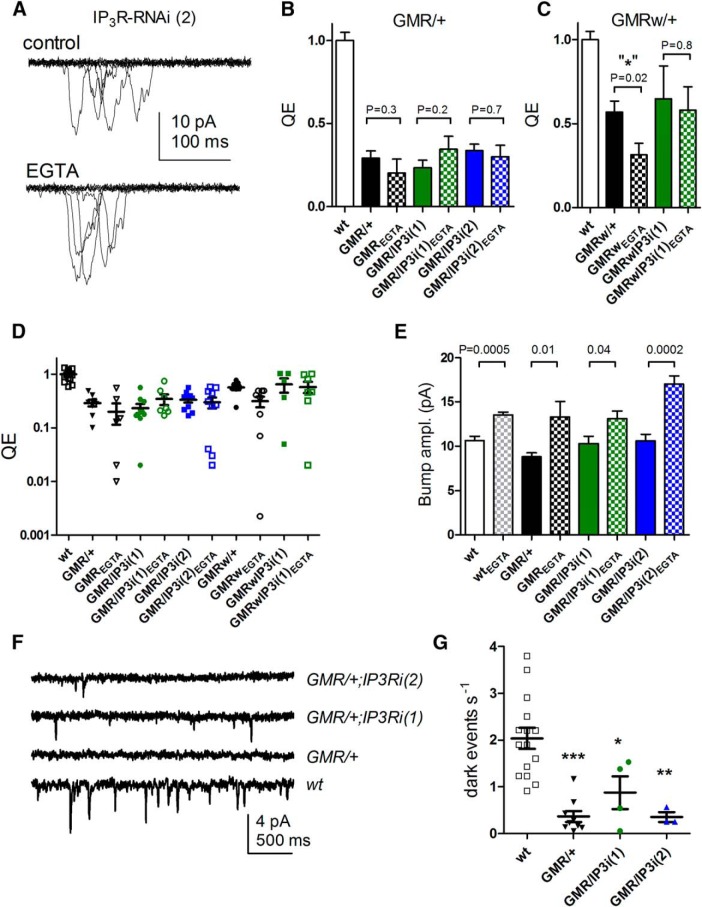
*GMRGal4* phenotypes in whole-cell recordings of light-induced currents. ***A***, Quantum bumps (10 superimposed traces, including “failures”) in response to dim flashes containing on average ∼0.5 effective photons recorded from *GMR/+;IP_3_R-RNAi* (two copies) photoreceptors with control electrode solution and with 1 mm EGTA (below). Note larger amplitudes with EGTA electrode (see also ***E***). ***B***, ***C***, QE determined from such recordings, normalized to wild-type (*n* = 20), in flies expressing one copy of *GMRGal4* (*GMR*/+ or *GMRw/+*) with or without *UAS*-*IP_3_R-RNAi* (one or two copies) and with or without 1 mm EGTA in the electrode (mean ± SEM, *n* = 5–10 cells per condition; see ***D***). All lines with one copy of GMRGal4 had reduced QE compared with wild-type (*p* < 0.001, one-way ANOVA, Dunnett’s multiple comparison), but inclusion of EGTA made little or no difference: one exception (in ***C***) was in flies with one copy of *GMRw* but without *IP_3_R-RNAi*. This was marginally significant (*p* = 0.02) with a direct *t* test, but not with a one-way ANOVA including data with and without EGTA from *GMRw*/*IP_3_R-RNAi* flies as well. ***D***, Same data showing QE in all cells on log_10_ plot: note variability in all *GMRGal4/+* backgrounds (total *n* = 78 cells): whereas most cells had QE two- to fourfold lower than in wild type, 11 cells had ≥10-fold lower QE. ***E***, Bump amplitudes were larger in recordings made with 1 mm EGTA in the electrode in all backgrounds (mean ± SEM of average bump amplitudes from *n* = 4–10 cells, each with 30–100 bumps). ***F***, Dark noise recorded with standard electrode solution (no EGTA). In wild-type cells, spontaneous ∼2-pA events occur at rates of ∼2/s, but backgrounds with one copy of *GMRGal4* (with or without *IP_3_R*-*RNAi*) showed far fewer events. ***G***, Summary of data: all lines with one copy *of GMRGal4* (*GMR*/+) had significantly fewer dark events than wild type (*, *p* < 0.05; **, *p* < 0.01; ***, *p* < 0.001), but there was no significant effect of one or two copies of *UAS*-*IP_3_R*-*RNAi* (one-way ANOVA with Tukey’s posttest).

In whole-cell recordings, sensitivity can be defined by the relative quantum efficiency (QE), i.e., the fraction of incident photons evoking a quantum bump, which is determined by the amount of visual pigment (rhodopsin) and the probability that a photoisomerized rhodopsin successfully generates a quantum bump. One of the key findings in Kohn et al. (2015) was that QE was approximately twofold reduced in *GMRGal4/+*;*IP_3_R*-*RNAi* flies compared with wild-type flies, but only when the electrode solution was buffered with EGTA. When we attempted to confirm this, we found that QE was already reduced by approximately two- to threefold compared with wild-type in all backgrounds with one copy of *GMRGal4*, irrespective of *IP_3_R*-*RNAi*. However, QE was largely unaffected by the inclusion of EGTA in the electrode (Fig. 3*A–D*). One apparent exception were *GMRw*/+ controls (i.e., with one copy of *GMRGal4*, *UAS-wRNAi* but without *UAS*-*IP_3_R*-*RNAi*), which appeared to show an approximately twofold reduction in QE in recordings made with EGTA (*n* = 8). This was marginally significant (*p* = 0.02) on a direct *t* test, but not on an ANOVA including *GMRw/IP_3_R-RNAi* data with and without EGTA (*p* = 0.18). In all lines, inclusion of EGTA in the pipette increased bump amplitude, presumably due to suppression of the negative feedback effects of Ca^2+^ on the bump wave form (Fig. 3*E*). This was also reported by Kohn et al. (2015) and confirms the effectiveness of EGTA in our experiments.

A conspicuous feature of recordings from flies carrying one copy of *GMRGal4* was a large variability in QE (relative SD ∼0.7, vs. ∼0.2 for wild type). Although the majority of cells had QE values clustering within ∼20–60% of wild-type values, cells were regularly encountered (11/78 cells) in which QE was at least 10-fold, and sometimes >100-fold, lower (Fig. 3*D*). Such large variations are not encountered in recordings from wild-type adult photoreceptors, though they are a feature of recordings from pupal photoreceptors (Hardie et al., 1993). Although numbers were too small for evaluation of statistical significance, we noted that most (8/11) of these conspicuously insensitive cells, but not all of them, were recorded with EGTA-containing electrodes; however, *IP_3_R*-*RNAi* appeared not to make a difference (5/11 cells were from *GMR* or *GMRw* controls without *IP_3_R*-*RNAi*). In certain experiments (e.g., Sr^2+^ substitution; see further below), Kohn et al. (2015) reported large reductions in sensitivity in *IP_3_R*-*RNAi* flies when recorded with EGTA. Given that we were unable to replicate these results even using IP_3_R-null mutants (see below), one possible explanation for their findings is the fortuitous inclusion of data from such insensitive cells.

Using standard electrode solutions (without EGTA), whole-cell recordings from wild-type photoreceptors exhibit an ongoing spontaneous barrage of miniature (∼2-pA) bump-like events occurring at rates of ∼2/s, and which are believed to arise from spontaneous activation of G proteins (Hardie et al., 2002; Elia et al., 2005; Chu et al., 2013). Kohn et al. (2015) reported that *GMRGal4*/*IP_3_R*-*RNAi* flies had reduced levels of spontaneous activity even without EGTA in the electrode. However, we found that these spontaneous dark events were greatly reduced in frequency in *GMRGal4*/+ photoreceptors irrespective of *IP_3_R*-*RNAi* (Fig. 3*F*, *G*), suggesting that this result may also be attributable to *GMRGal4* expression rather than IP_3_R knockdown.

In summary, flies expressing one copy of *GMRGal4* displayed a number of phenotypes suggestive of compromised development. These are presumably caused by adverse, nonspecific effects of Gal4 in the developing eye and seem potentially able to account for many, if not all, of the results reported by Kohn et al. (2015). We did not, however, detect any additional effects of *IP_3_R*-*RNAi* knockdown using either one or two copies of *UAS-IP_3_R-RNAi*. Unfortunately, the *GMRGal4,UAS-wRNAi* fly stock used by Kohn et al. (2015) has been lost, so we cannot rigorously exclude the possibility that their *IP_3_R*-*RNAi* flies would still have shown some phenotypes when compared with appropriate *GMRGal4* controls. Therefore, for further analysis, we turned to IP_3_R-null mutants (Raghu et al., 2000b), reasoning that these would show significantly more severe phenotypes, should any exist.

### Null mutants of the InsP_3_ receptor (*itpr*)

Null mutations of the IP_3_R (*l(3)itpr^90B.0^*, referred to as *itpr*) are larval-lethal; however, whole-eye null mosaics can be generated by inducing mitotic recombination in the developing eye using the flippase-flippase recognition target (FLP-FRT) system under control of *ey-Gal4* (Stowers and Schwarz, 1999; Raghu et al., 2000b). As controls, we used wild-type flies, *itpr/+* heterozygote siblings from the same cross used to generate the eye mosaics, and “wild-type” mosaic eyes generated using an otherwise wild-type FRT chromosome (see Materials and Methods). Previously, we found that *itpr*-null eye mosaics expressed no detectable IP_3_R protein and only trace amounts of genomic DNA or mRNA, from contaminating material in the dissections used to isolate retinal tissue (Raghu et al., 2000b). Because these flies had been left in stock for more than ten years, we first checked the genotype by genomic qPCR and confirmed that the IP_3_R gene was reduced to trace amounts (15.7 ± 1.8%, *n* = 3, vs. control wild-type retinae), attributable to contaminating (nonretinal) tissue in dissected retinae (Raghu et al., 2000b). We also confirmed the virtual absence of any mRNA in retinal tissue by qRT-PCR (13.1 ± 3% IP_3_R mRNA remaining in sample compared with wild-type controls, *n* = 3).

### ERGs of *itpr*-null mosaic eyes show a variable phenotype

**Figure 4.**
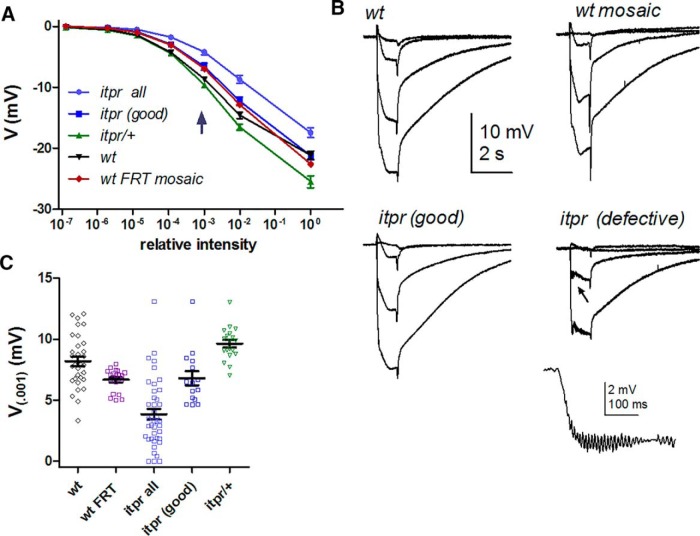
ERGs in *itpr*-null mosaic eyes. ***A***, Response intensity (*V/log I*) functions measured from ERG (plateau at end of 1-s stimuli) in *itpr*-null mosaics (all flies, *n* = 40), selected *itpr* mosaics without obvious ERG defects (*itpr* “good,” *n* = 15), wild-type (*n* = 32), *itpr*/+ sibling controls (*n* = 18), and “wild-type” FRT mosaic eyes (*n* = 18). ***B***, Representative ERG traces, including examples of a “good” and an obviously defective ERG from *itpr* mosaics: inset shows trace with oscillations (arrow) on expanded scale. ***C***, Scatter plot of ERG amplitudes to relative intensity 10^−3^ (arrow in ***A***). Overall, *w^+^*; *itpr* mosaic flies showed a significant reduction in amplitude (*p* ∼10^−9^ vs. wt, *p* = 0.0001 vs. wt FRT mosaic; two-tailed unpaired *t* test). If the obviously compromised recordings (*V*_(0.001)_ <4 mV, oscillations and reduced synaptic transients) were excluded (*itpr* “good”), ERG amplitudes were still reduced compared with wild type and *itpr*/+ controls but now similar (*p* = 0.8) to recordings from wild-type mosaic eyes generated using an otherwise wild-type FRT82B chromosome (wt FRT).

Previously, it was reported that photoreceptor responses from *itpr* eye mosaics were indistinguishable from controls in intracellular recordings (Acharya et al., 1997) or whole-cell recordings (Raghu et al., 2000b). However, at that time, no measurements were made using ERG recordings. When we measured ERGs from *itpr* mosaic eyes, amplitudes did in fact appear significantly reduced whether compared with wild-type, *itpr*/+ sibling controls, or wild-type mosaic eyes (*p* < 10^−5^, Fig. 4). However, it was also apparent that there was considerable variability in the ERGs. In ∼40% of flies (15/40), the ERGs resembled those from control flies; however, in others, the ERG was clearly compromised, typically showing reduction of “on” and “off” transients and development of oscillations, which are generally considered to reflect defects at the level of the synapse. If these obviously compromised ERGs were excluded from the analysis, the ERG amplitudes were still reduced compared with wild-type or *itpr*/+ controls (*p* = 0.047), but not compared with recordings from wild-type mosaic eyes (*p* = 0.8, Fig. 4). By implication, ERGs from “wild-type” mosaic eyes were also significantly less sensitive than wild type controls (*p* = 0.005).

**Figure 5.**
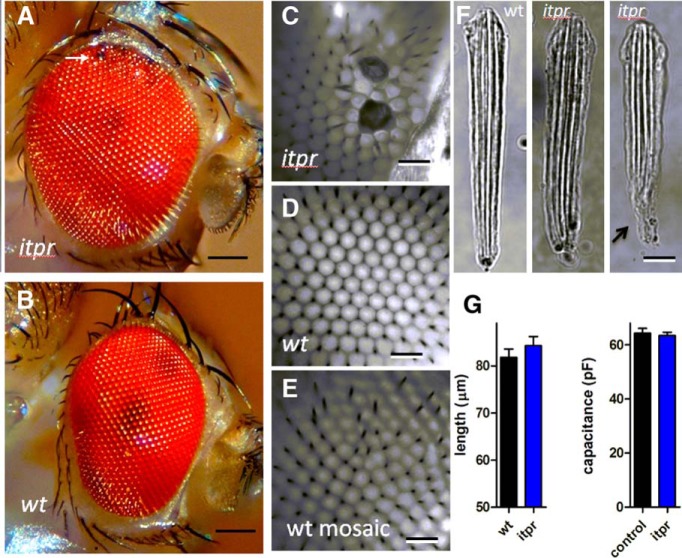
Structural abnormalities in *itpr* mosaic eyes. ***A***, *itpr* mosaic eyes were noticeably larger and rounder in appearance than wild type (***B***, ***D***) and frequently had areas of irregular and/or blackened facets (detail in ***C***, ***D***). ***E***, Wild-type mosaic eyes (generated using an otherwise wild-type FRT chromosome) also sometimes showed irregular facets, but not the blackened facets or bulbous appearance of *itpr* mosaic eyes. ***F***, Most dissociated ommatidia from *itpr* mosaic eyes appeared wild-type–like in appearance, but characteristically many still retained some of the axon terminal (arrow, right), which was almost invariably broken off in preparations from wild-type eyes. ***G***, Ommatidial lengths and whole-cell capacitances in *itpr* mosaics were similar to those of controls (control = wild-type and *itpr*/TM6 pooled; mean ± SEM, *n* = 11–26 ommatidia/cells). Scale bars; (***A***, ***B***) 80 µm, (***C–E***) 30 µm, (***F***) 10 µm.

**Figure 6.**
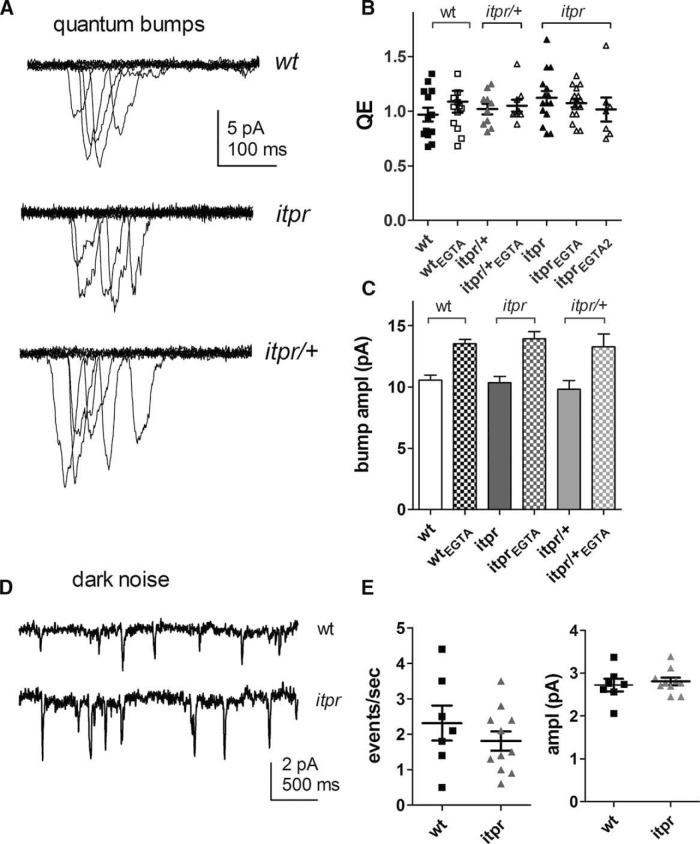
Whole-cell recordings from *itpr*-null mosaics. ***A***, Quantum bumps: 10 superimposed traces (including failures) from responses to 1-ms flashes containing on average ∼0.5 effective photons, recorded with 1 mm EGTA in electrode in wild-type, *itpr* mosaic null, and *itpr/+* heterozygotes (sibling controls). ***B***, Summary of QE in recordings with and without EGTA, normalized to wild-type values. There was no significant effect of EGTA or *itpr*-null mutation (mean ± SEM; *itpr* control electrode solution, *n* = 15 cells; *itpr* recorded with 1 mm, *n* = 15; 2 mm EGTA, *n* = 7; wt and *itpr*/+ controls, 9–14 cells; *p* = 0.77, one-way ANOVA). ***C***, Mean bump amplitude was increased in recordings made with EGTA (*n* = 4–10 cells). ***D***, Dark noise recorded in wild-type and *itpr* mutants recorded using normal electrode solution (no EGTA); both showed similar levels of dark noise. ***E***, Dark event rates and amplitudes (mean ± SEM) in wild-type (*n* = 7 cells) and *itpr* mosaics (*n* = 11 cells) were similar (*p* = 0.34 for rates and 0.64 for amplitude, two-tailed *t* test).

**Figure 7.**
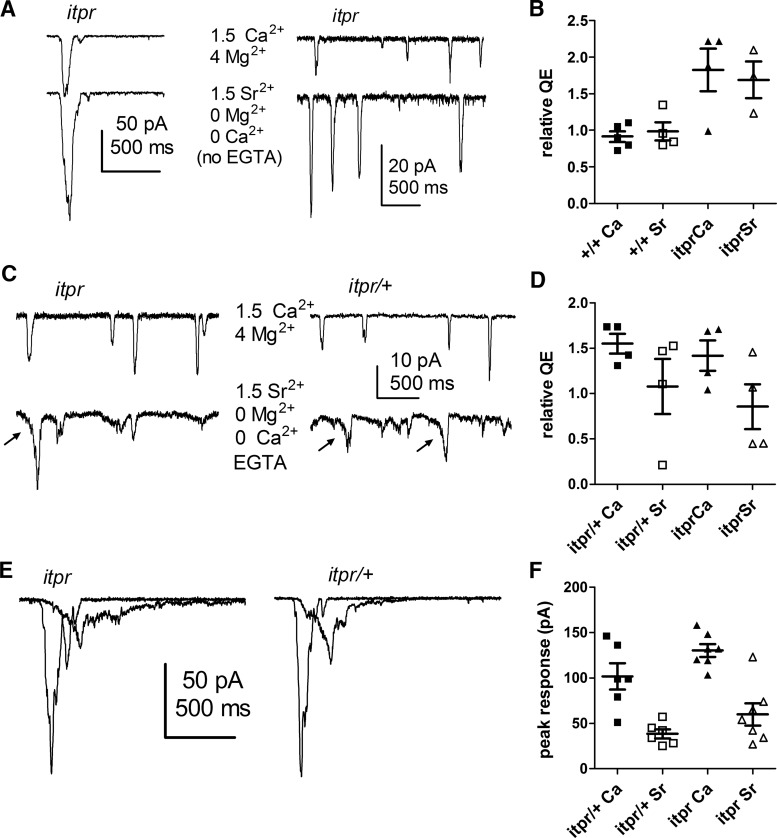
Sr^2+^ substitution affects wt and *itpr* mosaics similarly. ***A***, Substitution of control bath (1.5 Ca^2+^, 4 Mg^2+^ upper traces) with nominally Ca^2+^- and Mg^2+^-free solution and 1.5 mm Sr^2+^ without EGTA in the bath (lower traces from same cells). Left, response to 1-ms flashes containing ∼25 effective photons; right, quantum bumps in response to continuous dim light from *itpr*-null photoreceptor cells recorded using EGTA (1 mm) in the electrode. ***B***, Summary of QE data from *itpr*-null mosaics and wild-type controls (+/+). ***C–F***, Bath substitution with EGTA buffered Sr^2+^ (1 mm EGTA, nominally 0 Ca^2+^, 0 Mg^2+^ and 2.5 mm Sr^2+^: free [Sr^2+^] = 1.5 mm). Upper traces, before; bottom traces, same cells after perfusing with EGTA-buffered Sr^2+^ solution. ***C***, Quantum bumps elicited under these conditions showed defects in both controls (*itpr*/+) and null mutants (*itpr*), often showing amplification only after a slow ramping phase (arrows). ***D***, However, QE was only slightly affected, with no discernible difference between *itpr* and *itpr*/+ controls. ***E***, Macroscopic responses to 1-ms flashes (∼25 effective photons) under the same conditions (slower traces during 0 Ca^2+^, 0 Mg^2+^ 1.5 Sr^2+^ plus EGTA perfusion). ***F***, Peak amplitudes of responses were similarly affected in *itpr* and *itpr*/+ controls.

**Figure 8.**
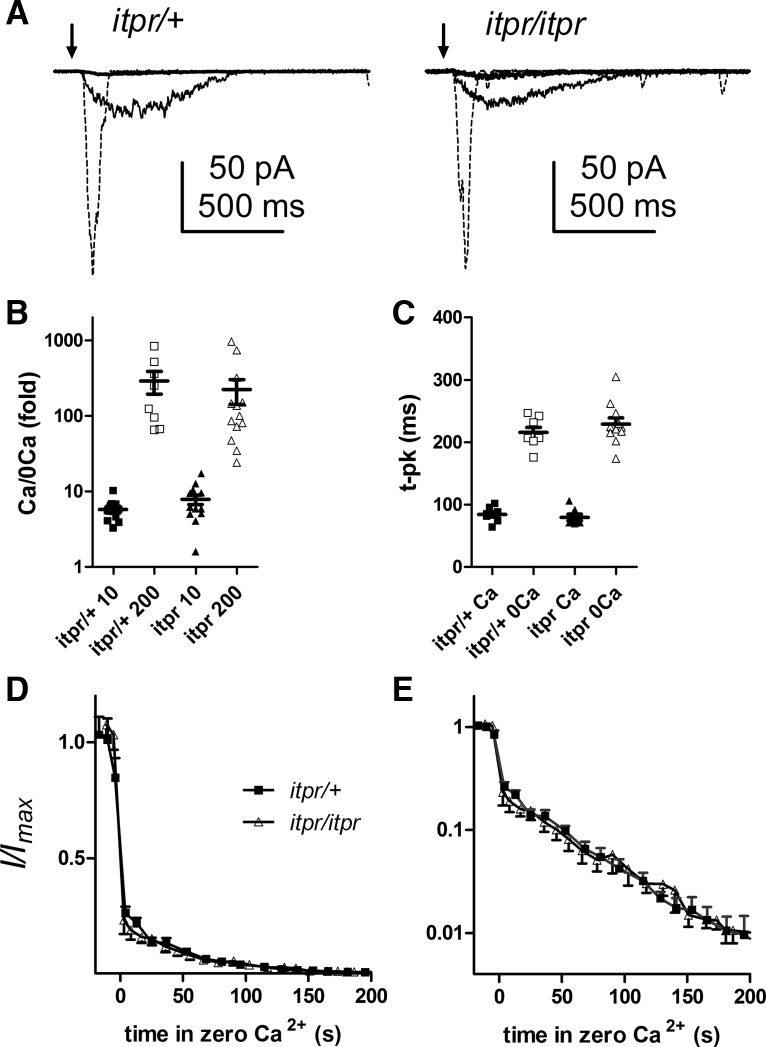
Effects of Ca^2+^-free perfusion in *itpr*-null photoreceptors are similar to those of controls. ***A***, Responses to brief flashes containing ∼25 effective photons recorded with EGTA-buffered electrode solutions before (dotted traces), 10 s after (larger slow responses), and 200 s after perfusing with Ca^2+^-free solution (1 mm EGTA). ***B***, ***C***, The reduction in peak amplitude (log_10_ scale) after 10- and 200-s perfusion and slowing of the response (measured by time to peak after 10- to 60-s perfusion) were similarly affected in *itpr*-null mosaic and *itpr*/+ sibling controls (*p* = 0.2–0.7, two-tailed *t* tests). ***D***, Time course of suppression of the light response in Ca^2+^-free solution was similar in *itpr*-null mosaics and controls (mean ± SEM, *n* = 9–12 cells). ***E***, Same data plotted on log_10_ scale.

In addition to these variable defects in the ERG, the outward appearance of *itpr* mosaic eyes revealed clear abnormalities, usually being noticeably larger and more bulbous than wild-type eyes and typically containing a variable number of irregular or darkened facets (Fig. 5). “Wild-type” mosaic eyes occasionally also showed irregular facets, but were not noticeably different in shape or size and never showed the “scorched” facets typical of many *itpr* mosaic eyes. As described below, we were unable to detect any *itpr* phenotypes at the level of the photoreceptors in whole-cell recordings (Figs. 6–8) or with completely noninvasive *in vivo* Ca^2+^ imaging (see Fig. 10). Hence, we suggest that the variable ERG phenotype reflects defects in the overall structure of the eye, possibly owing to a role of the IP_3_R during development. For example, ERG amplitude is critically dependent on resistance barriers between the retina, lamina, and hemolymph (Heisenberg, 1971) and if short-circuited, even in a limited region, can be expected to have a potentially severe impact on the ERG. An indication of just such a defect was noted when preparing retinal tissue from freeze-dried heads (see Materials and Methods). In wild-type eyes, the retina separates cleanly and readily from the underlying neuropil (lamina) at a fracture plane near the base of the retina (Matsumoto et al., 1982); however, in *itpr* mosaics, this separation was noticeably more difficult to achieve, indicating structural differences at or around the base of the retina where the resistance barrier resides (Heisenberg, 1971). Probably related to this, in preparations of dissociated ommatidia from *itpr* mosaic eyes, ommatidia were frequently seen that had retained part of the axon terminal, whereas in wild-type preparations these are almost invariably broken off at the base of the retina (Fig. 5*F*).

### Whole-cell recordings: quantum efficiency and dark noise are unaffected in *itpr* mutants

Despite abnormalities in the overall eye structure, and in contrast to ommatidia from *GMRGal4/+* flies, dissociated ommatidia from *itpr* mosaic eyes had an essentially wild-type appearance (apart from the occasional retention of some axon terminal) and were of normal length, and in whole-cell recordings, the photoreceptors had capacitances and potassium channel profiles (not shown) similar to wild-type (Fig. 5*G*; compare Fig. 1).

The central argument of Kohn et al. (2015) was that, although recordings from *IP_3_R*-*RNAi* flies made with electrode solutions lacking Ca^2+^ buffers showed normal light responses, phenotypes, including a twofold reduction in QE, became apparent when using electrode solutions buffered with 1 mm EGTA. However, we found that the QE of *itpr*-null mosaic photoreceptors recorded with normal electrode solution was indistinguishable from QE in recordings made with electrode solution containing 1 mm or even 2 mm EGTA. Neither were there significant differences in QE between *itpr*-null and wild-type or *itpr*/+ heterozygote controls with or without EGTA in the electrode (Fig. 6*B*). As with *GMRGal4*/+ flies, we confirmed the larger bump amplitudes recorded using EGTA-buffered electrode solutions (Fig. 6*C*).

The only phenotype of *IP_3_R*-*RNAi* flies reported by Kohn et al. (2015) in whole-cell recordings made with electrode solutions without EGTA was a reduction in the rate of spontaneous dark events (dark noise). Because we found that such a reduction was a feature of recordings from *GMRGal4*/+ irrespective of *IP_3_R-RNAi* (Fig. 3*F*, *G*), we also recorded dark noise in photoreceptors from *itpr*-null mutant mosaics. However, we found no difference in the dark noise between *itpr*-null mutants and wild-type controls (Fig. 6*D*, *E*), both of which showed spontaneous events of similar amplitudes (∼2 pA) at rates of ∼2 events/s as previously reported (Hardie et al., 2002; Elia et al., 2005; Chu et al., 2013).

### *itpr* mutants are not more profoundly affected by Sr^2+^ substitution than controls

Arguably, the most dramatic effect of *IP_3_R*-*RNAi* knockdown reported by Kohn et al. (2015) was a severe (∼100-fold) reduction in QE when using EGTA in the electrode and bath Ca^2+^ substituted with Sr^2+^. In preliminary experiments, using the same solutions as those authors (1 mm EGTA in electrode; 1.5 mm Sr^2+^, nominally Ca^2+^- and Mg^2+^-free, but no EGTA in bath), QE appeared to be unaffected in either control flies or *itpr*-null mosaics (Fig. 7*A*). However, quantum bump amplitudes (and macroscopic responses) in both mutant and control became significantly larger because of the relief of channel block by Mg^2+^ (Hardie and Mojet, 1995).

Trace levels of Ca^2+^ in nominally Ca^2+^-free, unbuffered solutions are typically on the order of a few micromoles, which might still provide sufficient Ca^2+^ influx to sustain some degree of positive and negative feedback. We therefore proceeded to buffer the external 0 Ca^2+^, 0 Mg^2+^ solution with 1 mm EGTA, while increasing total Sr^2+^ to 2.5 mm. Because EGTA’s affinity for Ca^2+^ (*K_d_* ∼200 nm) is approximately two orders of magnitude higher than for Sr^2+^ (∼30 µm), this should reduce trace Ca^2+^ to low nm levels while leaving ∼1.5 mm free Sr^2+^ (Xu-Friedman and Regehr, 2000). Under these conditions, bump amplification in both controls and *itpr*-null cells was significantly impaired after 1- to 2-min perfusion, leaving many bumps reduced in amplitude and with slow, irregular time courses. Bumps that still showed amplification had characteristically altered waveforms in both mutants and controls, with a slow ramping phase often apparent before the onset of rapid amplification (Fig. 7 *C*, *D*, arrows). A similar behavior has been observed in solutions containing reduced external Ca^2+^ or in cells buffered internally with BAPTA, and was attributed to the role of Ca^2+^ influx in the sequential positive and negative feedback that shapes the bump wave form (Henderson et al., 2000). Again, however, there was no noticeable difference between *itpr*-null and control cells, with both showing only a minor (<50%) reduction in QE. Because of the difficulty of unequivocally identifying bumps (and hence accurately estimating QE) under these conditions, we also simply recorded macroscopic responses to brief flashes. Peak amplitudes of responses were reduced approximately three- to fivefold after 2- to 3-min perfusion with the EGTA buffered Sr^2+^ solution, and time to peak slowed from ∼80 ms to >200 ms. Again, however, there was no difference between *itpr*-null mosaics and controls (Fig. 7*E*, *F*).

Previously, Katz and Minke (2012) reported that Sr^2+^ substitution still supported bump amplification but eliminated dark noise, and argued from this that there must be at least two sites for Ca^2+^-dependent facilitation (e.g., PLC and the channels). Although we do not exclude the possibility of two sites, we do not believe that this can be concluded from the effects of Sr^2+^. When trace Ca^2+^ is buffered with EGTA, our results indicate that Sr^2+^ influx is also much less effective than Ca^2+^ influx in supporting light-induced bump amplification (whether in controls or *itpr*-null mutants), and hence the most parsimonious explanation would be that the same site could also be responsible for the reduction in dark events.

### Responses under Ca^2+^-free conditions are not influenced by *IP_3_R*-null mutation

If Ca^2+^ release from IP_3_Rs is critical in facilitating phototransduction, then the simplest and most direct test for revealing its role should be testing sensitivity to light in Ca^2+^-free bath, an experiment not performed by Kohn et al. (2015). Previously, we reported that responses recorded in Ca^2+^-free bath were unaffected in *itpr*-null mosaics (Raghu et al., 2000b); however, in those experiments, the pipette solution would have contained trace Ca^2+^. We therefore repeated these experiments using 1 mm EGTA in the electrode. Within seconds of perfusing with Ca^2+^-free solution (also buffered with 1 mm EGTA) via a puffer pipette, peak responses were slowed and amplitude was reduced approximately fivefold, which is slightly more severe than the approximately threefold reduction previously reported with normal electrode solutions (e.g., Hardie, 1991a; Ranganathan et al., 1991; Reuss et al., 1997; Raghu et al., 2000b). With time, the sensitivity declined further, presumably as cytosolic Ca^2+^ levels reequilibrated to lower values, and after 3 min of perfusion, sensitivity (peak amplitude) was reduced by ∼100-fold (Fig. 8). However, the pronounced suppression seen under these conditions was similar, and followed a similar time course, whether recorded from control flies (*itpr/+* siblings) or *itpr*-null mosaics.

In summary, in whole-cell recordings from IP_3_R-null mutants (*itpr*), we were unable to detect any of the phenotypes described by Kohn et al. (2015) and attributed to IP_3_R knockdown in *IP_3_R*-*RNAi* flies. Furthermore, in contrast to the variability we encountered in ERG recordings from *itpr* mutants or whole-cell recordings from *GMRGal4* flies, we found no such variability in responses from single photoreceptors (with or without EGTA), despite making recordings from >60 cells from >30 flies (relative SD for QE ∼0.2–0.25 in both mosaics and controls). This reinforces our view that the variable ERG phenotypes in *itpr*-null mosaics (Fig. 4) are likely to reflect variable defects in overall eye structure or retinal resistance barriers rather than photoreceptor sensitivity.

### Responses in hypomorphic *norpA* (PLC) mutants are suppressed by *GMR-Gal4*, but unaffected by *IP_3_R-RNAi*

Although null mutants of PLC (*norpA*) have essentially no light response (Bloomquist et al., 1988), hypomorphic mutants—which still generate finite, albeit compromised, responses—can yield useful information on intermediate steps in the transduction cascade (e.g., Cook et al., 2000; Hardie et al., 2002). Kohn et al. (2015) reported that one copy of *IP_3_R*-*RNAi* driven by *GMRGal4* led to a further substantial reduction in the ERG in *norpA^H43^* mutant flies, which have ∼10-fold reduced PLC activity because of a point mutation in the catalytic site. This was attributed to the requirement of residual Ca^2+^ release from InsP_3_-sensitive stores to facilitate the weakened response in the PLC hypomorphic background (Kohn et al., 2015). However, controls from *norpA^H43^* bearing one copy of *GMRGal4* were again lacking. We therefore repeated these experiments, but now comparing white-eyed *norpA^H43^* flies (*norpA^H43^;bw;st*) with *norpA^H43^* flies carrying one copy of *GMRw* with and without *UAS*-*IP_3_R*-*RNAi*.

**Figure 9.**
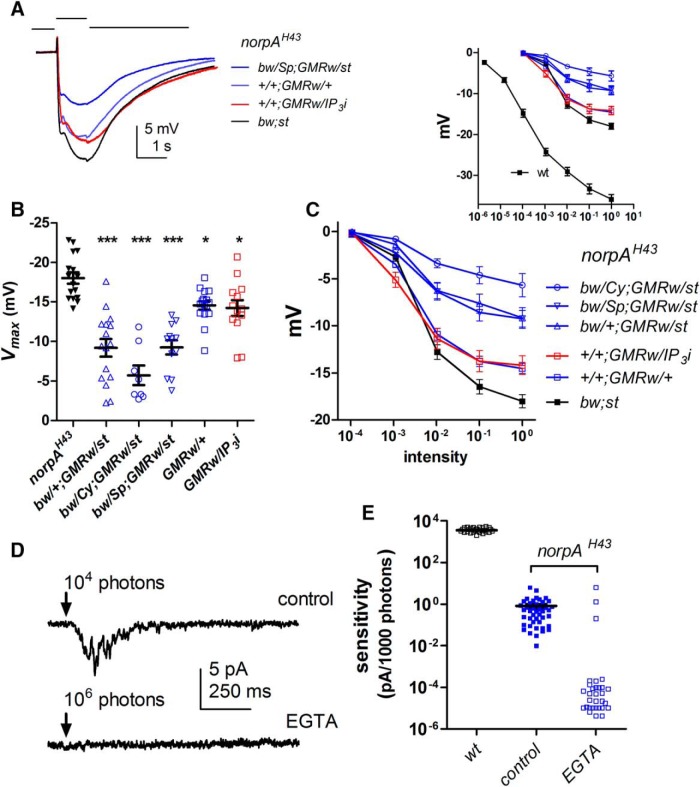
GMRGal4, but not *IP_3_R*-*RNAi*, suppresses sensitivity in *norpA^H43^*. ***A***, Representative ERG responses to 1-s flashes of submaximal intensity (10^−1^ on ***C***) in *norpA^H43^* mutant backgrounds. *norpA^H43^* flies carrying one copy of *GMRw* (generated by three independent crosses) had consistently smaller responses than *norpA^H43^*; *bw*;*st* control (without GMR). Flies also expressing *UAS*-*IP3R*-*RNAi* (red) had among the largest responses and were indistinguishable from their closest control (*norpA^H43^;+/+;GMRrw/+).* ***B***, ***C***, *V*/log *I* curve and *V_max_* values from all genotypes (mean ± SEM, *n* = 12–16 flies). *V_max_* in all backgrounds with one copy of *GMRGal4* were significantly (*, *p* < 0.05; ***, *p* < 0.001) suppressed compared with wild-type (one-way ANOVA, Dunnett’s multiple comparison test). ***D***, Whole-cell recordings from *norpA^H43^* photoreceptors. Top, response to 10-ms flash containing ∼10^4^ wild-type effective photons recorded with control electrode solutions. Bottom, a ∼100× brighter flash (10^6^ photons) elicited no response in a cell recorded with 1 mm EGTA. ***E***, Sensitivity (tested ∼2 min after establishing the whole-cell configuration) expressed in pA/1000 wild-type effective photons in *norpA^H43^* recorded with control electrode solution was approximately four orders of magnitude less than in wild-type (note log_10_ plot). With EGTA, there was no detectable response in 28 of 31 cells to flashes containing 10^6^ photons (the variation in amplitudes of these data points reflects noise in the baseline).

ERGs from *norpA^H43^* flies carrying one copy of the *GMRGal4*, *w-UAS-RNAi* chromosome (*GMRw*/+) appeared rather sensitive to genetic background. In the F1 of three different crosses to introduce one copy of *GMRw* into a *norpA^H43^* background (Fig. 9), ERG amplitudes were in each case significantly reduced compared with the parent *norpA^H43^;bw;st* control. However, *norpA^H43^*;+/+;*GMRw*/*UAS*-*IP_3_R-RNAi* flies actually had the highest sensitivity of any of the crosses and were indistinguishable from their most closely matched control (*norpA^H43^*;+/+;*GMRw*/+). The response amplitudes in *norpA^H43^*;*bw*/+;*GMRw*/*st, norpA^H43^*;*bw*/*Sp*;*GMRw*/*st*, or *norpA^H43^*;*bw*/*Cy*;*GMRw*/*st*, which represent the genotypes closest to *norpA^H43^*;*GMRw/bw; IP_3_R-RNAi/st* flies of Kohn et al. (2015), were more severely reduced—in fact, as severely as the data reported in their paper. Kohn et al. (2015) attributed this reduction in sensitivity to *IP_3_R*-*RNAi* knockdown, but according to our results it appears attributable to one copy of *GMRGal4*.

Kohn et al. (2015) also reported a pronounced effect of *IP_3_R*-*RNAi* in whole-cell patch-clamp recordings from *norpA^H43^*. Once again, in recordings made with electrode solution without EGTA, they reported no significant difference between *norpA^H43^* and *norpA^H43^*;*GMRw*/*IP_3_R*-*RNAi* flies, with both having a similar ∼4-log unit reduction in sensitivity compared with wild type. By contrast, when using EGTA-buffered electrode solution, *norpA^H43^* cells were reported to be unaffected, whereas sensitivity in *norpA^H43^*;*IP_3_R*-*RNAi* was further drastically reduced, such that cells were essentially completely unresponsive to the brightest lights. However, when we recorded from *norpA^H43^* photoreceptors (no *GMRGal4* and no *IP_3_R-RNAi*) we found that 1 mm EGTA in the electrode already eliminated or drastically reduced sensitivity to light (Fig 9*D*, *E*). Thus, with control electrode solutions (no EGTA), all *norpA^H43^* photoreceptors responded to flashes containing ∼10^4^ effective photons with small but robust responses. With EGTA in the electrode, however, the majority of cells (28/31) gave no response at all to 100× brighter flashes (∼10^6^ photons).

In summary, we found that one copy of *GMRGal4* significantly suppressed the ERG in *norpA^H43^* but found no additional effect of *IP_3_R*-*RNAi*, whereas in whole-cell recordings, we already found a profound suppression of sensitivity by EGTA in *norpA^H43^*. It is difficult to explain the failure of Kohn et al. (2015) to find an effect of EGTA in *norpA^H43^* control flies. However, we found that *norpA^H43^* photoreceptors can be very sensitive to facilitation, and the few cells (3/31) that failed to show suppressed sensitivity using EGTA in the electrode had a substantial leak currents or low-resistance gigaseals, both likely permeable to Ca^2+^.

### Ca^2+^ “release” is not affected by InsP_3_ receptor mutation or RNAi

Measurements using fluorescent Ca^2+^ indicators in dissociated ommatidia show that the Ca^2+^ signal in response to blue excitation light (a supersaturating stimulus) is dominated by massive Ca^2+^ influx via light-sensitive channels (Peretz et al., 1994; Ranganathan et al., 1994; Hardie, 1996). In Ca^2+^-free bath, there is a smaller and slower rise in the fluorescent signal, of uncertain origin. Previously, using a ratiometric Ca^2+^ indicator dye (INDO-1), this residual “Ca^2+^-free” signal was reported to be unaffected in *itpr*-null mosaic mutants, suggesting it was not due to InsP_3_-induced Ca^2+^ release from internal stores (Raghu et al., 2000b). However, Kohn et al. (2015) reported that Ca^2+^ signals in Ca^2+^-free bath measured in ommatidia expressing GCaMP6f were further substantially reduced and slowed in *IP_3_R*-*RNAi* flies and concluded they were indeed due to InsP_3_-induced Ca^2+^ release.

**Figure 10.**
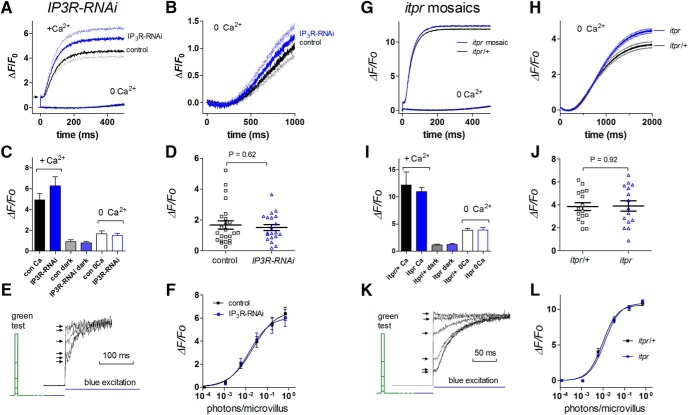
GCaMP6f signals are unaffected in *IP_3_R*-*RNAi* and *itpr* mutant flies. ***A***, Average traces of GCaMP6f fluorescence in the presence (1.5 mm) and absence of Ca^2+^ (perfusion from puffer pipette with 0 Ca^2+^ 1 mm EGTA) from dissociated ommatidia from flies expressing *GMRGal4;UAS-GCaMP6f* and two copies of *UAS*-*IP_3_R*-*RNAi* (mean, *n* = 15) and control (*GMRGal4*;*UAS*-*GCaMP6f* alone; *n* = 9 ommatidia); pale traces indicate SEM. Δ*F*/*F*_0_ values for both +Ca^2+^ and 0 Ca^2+^ traces were based on *F*_0_ values in Ca^2+^-free solution. ***B***, Ca^2+^-free responses on expanded scale. ***C***, Summary of Δ*F*/*F*_0_ values measured 1 s after light onset, as well as the dark-adapted level in the presence of Ca^2+^ estimated from the “pedestal” (arrow in ***A***). ***D***, Ca^2+^-free Δ*F*/*F*_0_ values replotted, showing all data points: there was no significant difference (*p* = 0.62, two-tailed unpaired *t* test) between control and *IP_3_R*-*RNAi* flies. ***E***, Two-pulse paradigm to determine intensity dependence of GCaMP6f signal *in vivo* from the deep pseudopupil (representative raw traces). Blue excitation was used to measure instantaneous GCaMP6f signal (arrows) in response to green (540 nm) test flashes (2 ms) of variable intensity delivered 300 ms earlier. ***F***, Resulting intensity dependences of GCaMP6f signal in *IP_3_R*-*RNAi* (two copies) and control flies (*GMR*/+; *UAS*-*GCaMP6f*) were essentially identical (mean ± SEM, *n* = 8 flies). ***G–L***, Similar data from *itpr*-null mosaics and sibling controls (*itpr*/+) expressing GCaMP6f under direct control of the Rh1 promoter (*ninaE*-*GCaMP6f*): *n* = 10–15 ommatidia/flies. No significant effects of the *itpr*–null mutation were detected.

We repeated these measurements using both *GMRGal4;UAS*-*GCaMP6f* with and without *IP_3_R*-*RNAi,* as well as *ninaE-GCaMP6f* under direct control of the Rh1 promoter (Asteriti et al., 2017) expressed in *itpr*-null mosaics. Our results from *itpr* mosaics are also reported elsewhere (Asteriti et al., 2017) but are replotted here with different controls (*itpr*/*TM6* siblings) for a comprehensive picture (Fig. 10*G–J*). Compared with responses in normal bath, responses under Ca^2+^-free conditions were reduced in amplitude and much slower, with a delay of ∼200 ms before any measurable increase in fluorescence. However, these Ca^2+^-free responses were at least as large and had a similar time course in flies with *IP_3_R-RNAi* (two copies) or *itpr*-null mutations (Fig. 10). Resting Ca^2+^ levels in the dark in the presence of extracellular Ca^2+^, estimated from fluorescence during the brief 10-ms latent period before any Ca^2+^ rise (Fig 10*A*, *C*, arrows), were also not significantly affected by *IP_3_R*-*RNAi* or *itpr*-null mutation.

Although our measurements in the presence of Ca^2+^ closely resembled those of Kohn et al. (2015), our signals recorded in Ca^2+^-free solutions were slower than they reported in control ommatidia, more closely resembling their responses in *IP_3_R*-*RNAi* flies. On the rare occasions that we did see a more rapid Ca^2+^ signal, it was immediately clear that it was due to failure to adequately perfuse the ommatidium with Ca^2+^-free solution, and we can only speculate that a similar explanation may account for the signals recorded by Kohn et al. (2015), who used whole-bath perfusion with a lower concentration (0.5 mm) of EGTA.

We also measured the Ca^2+^ rise *in vivo* in completely intact flies by monitoring GCaMP6f fluorescence in the deep pseudopupil (Asteriti et al., 2017). By using a two pulse paradigm, this allows accurate determination of the intensity dependence of Ca^2+^ rises in response to brief flashes of dimmer, physiologically relevant intensities (Fig. 10*E*, *K*). The response intensity functions measured in this way should also provide a more direct measure of *in vivo* photoreceptor sensitivity than the complex signal of the ERG. Data from flies carrying two copies of *IP_3_R-RNAi* and *itpr*-null mosaics were indistinguishable from their relevant controls, although *GMRGal4* flies (irrespective of *IP_3_R-RNAi*) were somewhat less sensitive than wild type (Fig. 10*F*, *L*).

## Discussion

Despite extensive experiments, we were unable to detect any effect of RNAi knockdown or genetic elimination (*itpr*-null mosaic eyes) of the InsP_3_ receptor on the light response of *Drosophila* photoreceptors. An apparent exception was the compromised ERG in at least some *itpr*-null mosaic eyes (Fig. 4). However, as discussed above, we are of the opinion that this results from abnormalities in eye structure (e.g., retinal resistance barriers), possibly indicating a role for IP_3_R in eye development. In contrast, using a more direct *in vivo* measure of photoreceptor function (live imaging of GCaMP6f in the DPP), we found no effect of *IP_3_R-RNAi* or *itpr*-null mutation on photoreceptor sensitivity *in vivo* (Fig. 10*F*, *L*). We did, however, find a number of phenotypes attributable to one copy of *GMRGal4* (Figs. 1–3), including reductions in sensitivity, dark noise, potassium currents, cell size, and capacitance. In addition, a notable feature of whole-cell recordings from photoreceptors from flies carrying one copy of *GMRGal4* was a pronounced variability in sensitivity, with some cells showing massive (up to ∼100-fold) reductions in QE irrespective of *IP_3_R*-*RNAi* (Fig. 3*D*). These phenotypes, which are suggestive of compromised development, have the potential to explain many, if not all, of the results of Kohn et al. (2015).

Although a clear Ca^2+^ rise can be detected in the absence of external Ca^2+^, this was too slow (∼200-ms latency) to influence the onset of the electrical light response, which has a latency of <10 ms and peaks within ∼100 ms even under Ca^2+^-free conditions at these intensities (e.g., Huang et al., 2010). As previously reported using Ca^2+^ indicator dyes (Raghu et al., 2000b), we also found that this signal was unaffected by either *itpr*-null mutation or *IP_3_R*-*RNAi* knockdown (Fig. 10) and is therefore presumably not mediated via InsP_3_-induced release from internal stores. Previously, we found that this signal all but disappeared in the absence of extracellular Na^+^ and suggested that the rise might be due to reequilibration of Na^+^/Ca^2+^ exchange in response to the massive light-induced Na^+^ influx that persists under these conditions (Hardie, 1996). This was questioned by Cook and Minke (1999), who proposed that only extracellular Na^2+^, but not influx, was required for the Ca^2+^ rise in Ca^2+^-free solutions. However, in a recent study (Asteriti et al., 2017), we found that not only was this Ca^2+^-free rise dependent on Na^+^ influx, but it was also eliminated in mutants of the Na^+^/Ca^2+^ exchanger and accelerated by overexpression of the exchanger, strongly supporting our original suggestion.

In conclusion, we were unable to find any phototransduction phenotypes in *IP_3_R*-*RNAi* or *itpr*-null mutants either *in vivo* or in whole-cell recordings with or without EGTA in the electrode, and together with a recent study (Asteriti et al., 2017), we found no evidence for significant light and InsP_3_-induced release of Ca^2+^ from internal stores. Our results therefore support earlier conclusions that the IP_3_R plays no significant role in the light response in *Drosophila* photoreceptors (Acharya et al., 1997; Raghu et al., 2000b). We have however, described a number of significant photoreceptor phenotypes of *GMRGal4*/+ flies suggestive of compromised development, which we attribute to pleiotropic effects of Gal4 expression in the developing eye, and which should be carefully controlled for in any experiments making use of this widely used driver.

## Synthesis

Reviewing Editor: Anna Menini, SISSA (International School of Advances Studies) Decisions are customarily a result of the Reviewing Editor and the peer reviewers coming together and discussing their recommendations until a consensus is reached. When revisions are invited, a fact-based synthesis statement explaining their decision and outlining what is needed to prepare a revision will be listed below. The following reviewer(s) agreed to reveal their identity: Richard Payne

Both reviewers agree that this is a meticulous study that re-evaluates the role (or lack thereof) of the InsP3 receptor in Drosophila phototransduction. The manuscript convincingly disproofs a previous report that suggested a role for the InsP3 receptor in phototransduction. Minor concerns have been fully addressed.
